# The Problems of Venereal Disease

**Published:** 1921-03-05

**Authors:** 


**THE PUBLIC WELL-BEING**—(*continued*).

### The Problems of Venereal Disease.

As upheld on an earlier page, viewed as a whole, the report of the Special Committee on Venereal Disease appointed by the National Birth-Rate Commission is a wise and broad-minded document, which gives a fairly just proportion to the various elements of this difficult but pressing national problem. The Committee quite properly urge that very great emphasis should be laid on the importance of chastity, and they call upon parents, teachers, ministers of religion, social reformers and medical practitioners, legislators and administrators to combine in a national effort to assert this moral obligation. At the same time they face the immediate practical issue by asserting the necessity for adequate preventive measures; and, while they point out that if self-disinfection is carelessly or incompetently carried out it will afford no protection, and may even increase disease by encouraging a false sense of security, they make no pretence of under-estimating the importance of

**THE PUBLIC WELL-BEING**—*(continued)*.

placing the means within the reach of the public.

The medical profession is not likely to quarrel with the Committee's further conclusion that the public advertisement and sale by unauthorised persons of disinfectants or substances for the avowed purpose of preventing or treating venereal disease should be illegal. The refusal to recommend legislation making it obligatory for all persons contemplating marriage to produce health certificates will, on the other hand, not please Miss Nora March, and others who strenuously support her in this stringent demand. But it is as well to acknowledge that public opinion is unripe for so intimate a piece of State interference in the personal relations of the sexes, and that to fasten on a demand like this and to give it a false air of reality in a document of public interest and importance would be a waste of energy and merely lead to discredit the report in its more practical aspects. We confess that we attach little value to the pious opinion expressed by the Committee, as a kind of substitute for a certificate, that when a medical practitioner, a minister of religion, or a friend becomes aware of the possibility of an infected marriage, advice should be tendered the man or woman affected. There is certainly every probability that cases of infection will be more frequently and frankly divulged to medical practitioners in the future; but at such moments—the romantic moments immediately before marriage, when the coming event is known to all the friends of both parties, and the difficulty of withdrawal without a clear explanation very real—except in the case of a man or woman of strong will-power or highly developed conscience, good advice, even if it is not treated as a gratuitous impertinence, is usually wasted effort. It is quite possible, of course, that by a persistent appeal to the public conscience and the public intelligence, and by prudent and vigorous State effort through the local health centres, the disease which more than any other is sapping the vitality of modern civilisation may be controlled and finally stamped out without having recourse to drastic measures like the imposition of compulsory certificates of health before marriage, and even without compulsory notification. But we think it is not likely. The popular instinct is undoubtedly at present strongly against the first. But when in a few years' time the public mind has become familiar with an idea against which it now revolts, there may be a different tale to tell.

